# Prognostic value of RDW in cancers: a systematic review and meta-analysis

**DOI:** 10.18632/oncotarget.13784

**Published:** 2016-12-02

**Authors:** Linhui Hu, Manman Li, Yangyang Ding, Lianfang Pu, Jun Liu, Jingxin Xie, Michael Cabanero, Jingrong Li, Ru Xiang, Shudao Xiong

**Affiliations:** ^1^ Department of Hematology/Hematological Lab, The Second Hospital of Anhui Medical University, Hefei, Anhui, Peoples Republic of China; ^2^ Department of Physiology and Pathophysiology, School of Basic Medical Sciences, Fudan University, Shanghai, Peoples Republic of China; ^3^ University Health Network, University of Toronto, Ontario, Canada; ^4^ Department of Emergency, The Second Hospital of Anhui Medical university, Hefei, Anhui, Peoples Republic of China; ^5^ School of Nursing, Anhui Medical University, Hefei, Anhui, Peoples Republic of China

**Keywords:** red blood cell distribution width, prognosis, cancer, meta-analysis

## Abstract

Red blood cell distribution width (RDW), a parameter that used to differentiate the type of anemia for several decades, recent studies suggest it was a prognostic factor in various types of cancer patients. However, the prognostic value of RDW in cancer patients remains controversial. Here, we performed a meta-analysis and systematic review to evaluate the prognostic value of RDW in cancer patients. Relevant studies were picked out from the databases of Web of Science, Embase, Pubmed and Cochrane Library. A total of 16 papers with 4267 patients were included in this meta-analysis, and the combined results indicated that elevated RDW was associated with poor over survival (OS) (HR = 1.47, 95%CI:1.29-1.66), poor cancer-specific survival (CSS) (HR = 1.46, 95%CI:1.08-1.85), poor disease-free survival (DFS) (HR = 1.91, 95%CI:1.27-2.56), poor event-free survival (EFS) (HR = 2.98, 95%CI:0.57-5.39) and poor progress-free survival (PFS) (HR = 3.21, 95%CI:0.33-6.75) after treatment. Furthermore, the similar results were observed in subgroup analysis stratified by cancer type, cutoff value of RDW, sample size and ethnicity. In conclusion, this meta-analysis demonstrated that RDW may be a potential prognostic marker in patients with cancer, and high RDW may also be associated with poor outcomes.

## INTRODUCTION

Cancers are characterized by rapid progress and have now become a major cause of morbidity and mortality in most region worldwide [1], despite the development of effective drugs and supportive care, the majority of cancers are characterized by their incurability, low overall survival, and recurrence [2]. Given this, a lot of biomarkers were carried out to help with prognosis of cancer [3–5].

Red blood cell distribution width (RDW) is a parameter usually reported in a complete blood cell count panels that contains RDW-SD (RDW standard deviation) and RDW-CV (RDW coefficient of variation) value, and it reflects the size heterogeneity of red blood cells. For several decades, it has been used to analyze and discriminate the types of anemia in clinical practices [6]. Recently, RDW was considered as a inflammatory associated marker, and emerging studies suggested it was an potential factor for predicting overall mortality in a variety of human inflammation diseases [7, 8].

It is well known that Inflammation is a hallmark of cancer [9], accumulating studies have investigated the role of RDW in patients with various cancer, and RDW was proved to be an independent prognosis factor in lung cancer [10], prostate cancer [11], chronic lymphocytic leukemia [12] and so on. However, different comments came into view with increasing researches referring to RDW and cancer [13, 14], and the reliability of RDW acting as a prognostic biomarker in various malignancies is being challenged. Therefore, the prognostic value of RDW in cancer patients remains controversial. In comparison to the limitation of single study, meta-analysis can provide a useful tool for the detection of effects that may be missed by individual studies. To date, no meta-analysis has been carried out to identify the prognostic value of RDW in cancer patients. Here, to better understand the role of RDW in cancer patients, a meta-analysis and a systematic review are performed to assess the correlation between RDW and the survival outcomes in cancer patients.

## RESULTS

### Characteristics of included studies

**Figure 1:**
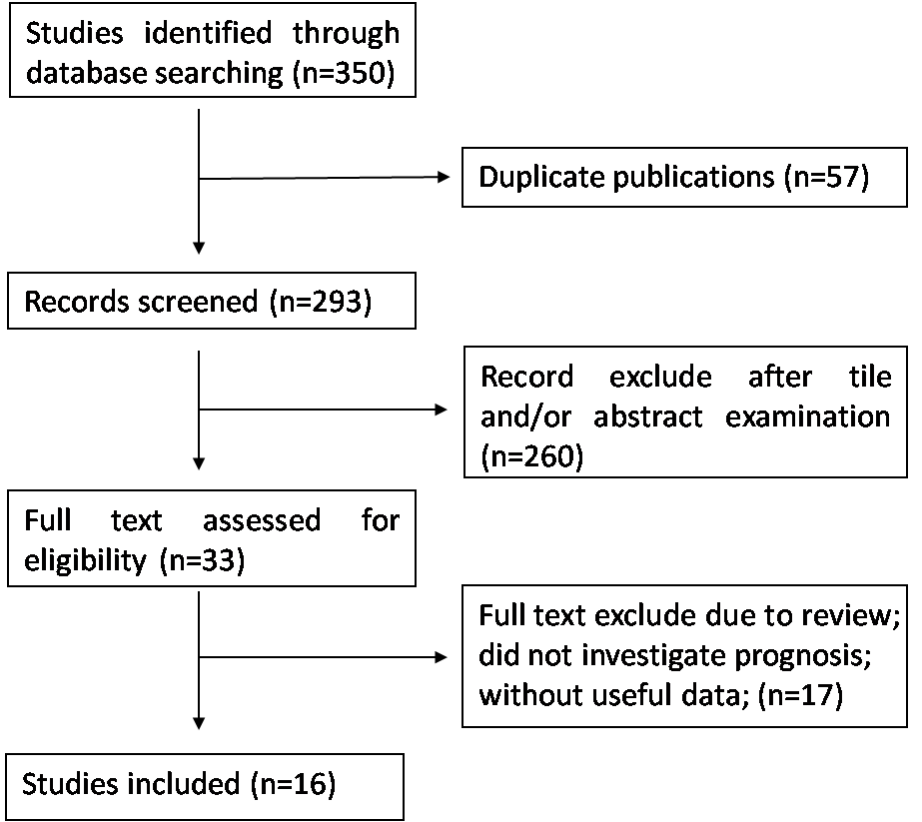
Flowchart presenting the steps of literature search and selection

As shown in Figure 1, a total of 350 articles meeting our included criteria were initially collected through our search strategy. After removed duplicate and irrelevant papers, a total of 16 articles were included in this analysis. For case ascertainment, fifteen articles [13–27] performed retrospective analysis (3953 participants), and one article [28] had both a retrospective design and a prospectively design in two independent patients population (314 participants). Therefore, a total of 16 articles including 17 studies with 4267 patients were included in this analysis with a median sample size of 179 (from 81 to 938). One study used RDW-SD for RDW [17] and others used RDW-CV. All studies were published between 2013 and 2016 in English peer-reviewed journals. These studies were from China, Japan, Italy, Korea, America, Turkey and Croatia, which evaluated various type of cancers, including four for esophageal cancer, three for lung cancer, two for hepatocellular carcinoma, two for breast cancer, one for multiple myeloma, one for diffuse large B-cell lymphoma, one for chronic myeloid leukemia, one for malignant mesothelioma, and one for upper tract urothelial Carcinoma.

3846 patients from fifteen studies reported OS, 616 patients from three studies reported CSS, 1096 patients from four studies reported DFS, 165 patients from two studies reported EFS and 146 patients from one studies reported PFS. Fourteen studies provided HR and 95% CI for OS, CSS, DFS and PFS, whereas three studies showed a survival curve for OS, one showed a survival curve for EFS, one showed a survival curve for DFS, and one summarized the total observed events and P value for DFS. The cutoff value of RDW in these studies was not uniform and ranged from 13.45 to 50. Fourteen studies used a cutoff value that was between 13.45 and 15, two studies used a cutoff value that was greater than 20. Age, tumor size and tumor stage at diagnosis are commonly investigated covariates that were adjusted for in Cox's proportional-hazard model evaluation of the relationship between the RDW and survival. The characteristics of included studies were listed in Table 1.

**Table 1:** The characteristics of included studies

First author	Year	Country	Ethnicity	Cancer type	Sample size	Outcome	Cutoff value	HR	NOS
Koma[19]	2013	Japan	Asian	Lung cancer	332	OS	15	Reported	6
Abakay[15]	2014	Turkey	Caucasian	Mesothelioma	152	OS	20	Reported	6
Lee[13]	2014	Korea	Asian	Multiple myeloma	146	OS, PFS	14.5	Reported	7
Yao[21]	2014	China	Asian	Breast cancer	608	OS, DFS	13.45	Reported	7
Chen[16]	2015	China	Asian	ESCC	277	CSS	14.5	Reported	7
Cheng[27]	2015	Taiwan	Asian	UTUC	195	OS, CSS	14	Reported	7
Iriyama[18]	2015	Japan	Asian	CML	84	OS, EFS	15	Estimated	7
Perisa[20]	2015	Croatia	Caucasian	DLBCL	81	OS, EFS	15	Reported	7
Smirne[28]	2015	Italy	Caucasian	Hepatocellular carcinoma	106 and 208	OS	14.6	Reported	8
Xie[25]	2015	America	Caucasian	Lung cancer	938	OS	15	Estimated	7
Hirahara[17]	2016	Japan	Asian	ESCC	144	CSS	50*	Reported	8
Huang[24]	2016	China	Asian	Breast cancer	203	OS, DFS	13.75	Reported	8
Kos[14]	2016	Turkey	Caucasian	Lung cancer	146	OS	14.2	Estimated	7
Sun[26]	2016	China	Asian	ESCC	362	OS	13.6	Reported	6
Wan[22]	2016	China	Asian	ESCC	179	OS, DFS	15	Reported	7
Zhao[23]	2016	China	Asian	Hepatocellular carcinoma	106	OS, DFS	14.5	Reported	7

Abbreviations: UTUC: upper tract urothelial carcinoma; CML: chronic myeloid leukemia; DLBCL: diffuse large B-cell lymphoma; ESCC, esophageal squamous cell carcinoma.

*: RDW was present as RDW-SD

### Meta-analysis results

**Figure 2:**
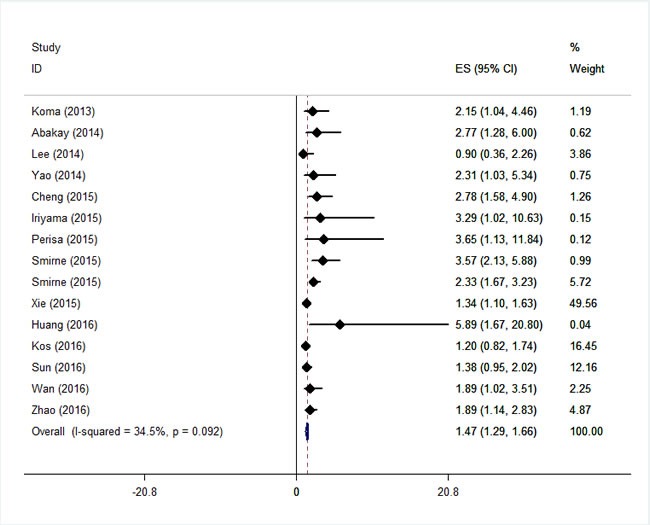
Forest plot for the association between RDW and the overall survival of patients with cancers

**Figure 3:**
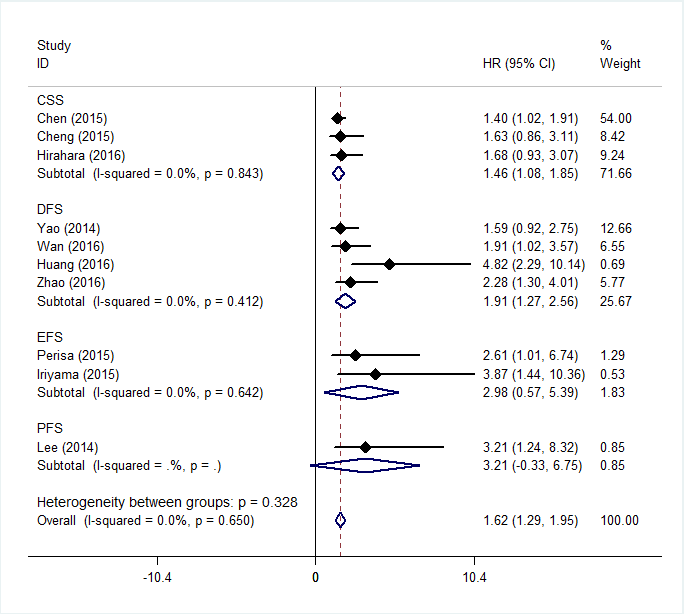
Forest plot for the association between RDW and the CSS, DFS, EFS and PFS of patients with cancer

As shown in Figure 2, the combined results of 16 studies showed elevated RDW was associated with poor OS (HR = 1.47, 95%CI: 1.29-1.66) with a small heterogeneity (I^2^ = 34.5%, P_heterogeneity_ = 0.092). Figure 3 summarized HR for CSS (HR = 1.46, 95%CI:1.08-1.85), DFS (HR = 1.91, 95%CI:1.27-2.56), EFS (HR = 2.98, 95%CI:0.57-5.39) and PFS (HR = 3.21 , 95%CI:-0.33-6.75), and there were no heterogeneity between the studies (I^2^ = 0, P_heterogeneity_ = 0.843; I^2^ = 0, P_heterogeneity_ = 0.412; I^2^ = 0, P_heterogeneity_ = 0.642).

Subgroup analysis for OS was also performed stratified by cancer type, sample size, cutoff value and ethnicity. As shown in Table 2, however, the summary HR remained significant in the subgroup.

**Table 2:** Subgroup analysis of the associations between RDW and overall survival

Subgroup	No. of studies	HR (95%CI)	*P*	Model	Heterogeneity	
					I^2^(%)	*P*
Caner types						
Hepatocellular carcinoma	3	2.25(1.70,2.80)	0.000	Fixed	24.3%	0.267
ESCC	2	1.46(0.97,1.95)	0.000	Fixed	0%	0.456
Breast cancer	2	2.49(0.38,4.59)	0.020	Fixed	0%	0.475
Lung cancer	3	1.32(1.09,1.56)	0.000	Fixed	0%	0.540
Hematologic malignancies	3	1.07(0.15,1.99)	0.023	Fixed	0%	0.399
Other	2	2.78(1.42,4.14)	0.000	Fixed	0%	0.993
Sample size						
<200	9	1.57(1.20,1.94)	0.000	Fixed	39.5%	0.104
≥200	6	1.46(1.23,1.68)	0.000	Fixed	35.1%	0.174
Cutoff value						
13≤ and >14	3	1.45(0.93-1.97)	0.000	Fixed	0%	0.471
14≤ and >15	6	1.87(1.20-2.53)	0.000	Random	65.1%	0.014
=15	5	1.39(1.14-1.65)	0.000	Fixed	0%	0.589
>15	1	2.77(0.41-5.13)	0.021	-	-	-
Ethnicity						
Caucasian	6	1.80(1.20,2.41)	0.000	Random	62.5%	0.021
Asian	9	1.59(1.23,1.96)	0.000	Fixed	0	0.494

Abbreviations: ESCC: esophageal squamous cell carcinoma; No.: number; HR: hazard ratio; CI: confidence interval. Random-effects model was employed when the p-value for heterogeneity test< 0.05

### Publication bias

**Figure 4:**
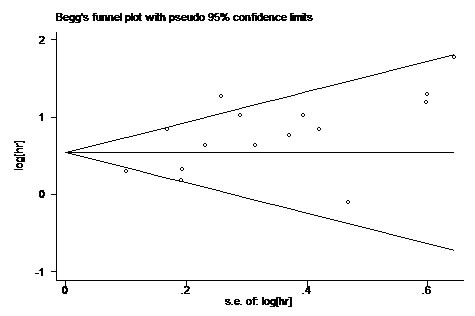
Begg's Funnel plot analysis of potential publication bias

**Figure 5:**
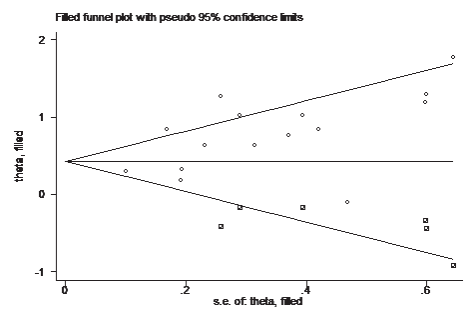
Funnel plot with trim and fill Circle represent identified studies, square represent estimated missing studies after adjustment for publication bias.

To assess publication bias in this study, the included studies were conducted by using Begg's funnel plots and Egger's test. The funnel plot for OS was asymmetry (Figure 4) and the result of Begg's (*P* = 0.138) and Egger's (*P* = 0.019) test indicated the possibility of publication bias. However, the pooled HR of 1.56 (95% CI, 1.26-1.99) obtained from trim and fill method was remained statistically significant with a symmetrical funnel plot (Figure 5), indicating that our results were robust and not affected by publication bias. Additionally, no publication bias was identified by Begg's and Egger's test for DFS (*P* = 0.308, *P* = 0.125) and CSS (*P* = 1.000, *P* = 0.121).

## DISCUSSION

This meta analysis included 17 studies with a total of 4267 patients to clarify the prognostic value of RDW in the pretreatment patients with cancer. The combined results indicated that elevated RDW significantly predicted poor OS, poor CSS, poor DFS, poor EFS and poor PFS of patients with cancer. Furthermore, the similar results were observed in subgroup analysis stratified by cancer type, cutoff value of RDW, sample size and ethnicity .To our knowledge, this was the first meta-analysis to explore the prognostic value of RDW in patients with cancers.

Cancer associated inflammation is recognized as a hallmark feature of tumor development and progression [9]. A lot of tumor secreted factors and cytokines secreted by inflammatory cells in the tumor microenvironment can influence the tumor cell proliferation, survival, drug resistance and migration. In the meanwhile, a variety of inflammation associated markers were carried out in the hope of developing cost-effective prognostic biomarkers in cancer patients [4, 5, 29]. RDW was found to have a strong, graded association with circulating high-sensitivity C-reactive protein (hsCRP) and erythrocyte sedimentation rate (ESR), which were the two most widely used plasma inflammatory biomarkers, in a large cohort of unselect adult outpatients [30]. Besides, in the healthy population, RDW correlated with inflammatory parameters such as plasma viscosity, ESR, fibrinogen, leukocyte and neutrophil counts [31]. And recently, RDW has emerged as a consistent and strong predictor of overall and disease-specific mortality in middle-age and older adults [8]. Based on these results, the prognostic value of RDW was investigated in a variety of cancer patients and gathering evidences suggested that RDW was an independent factor for prognosis [32, 33]. The mechanism remains unknown, but may be that, firstly, RDW correlated with IL-6, tumor necrosis factor-alpha, hepcidin and other circulating cytokines that can affect the tumor cell biological behavior [34, 35]; secondly, RDW may present the constitutive level of IGF-1 signaling, the critical factor accountable for metabolic aging and longevity [12]; thirdly, RDW showed the nutritional status of patients including iron, folate, and vitamin B12 [22], and lower RDW was associated with poor nutritional status which was another hallmark of cancer [36]. Furthermore, RDW is easily obtained from complete blood cell count panels, which is cost-effective, reproducible and automated. Thus, RDW is a promising prognostic inflammation marker helpful for the clinical decision-making process regarding cancer outcomes.

Nevertheless, several limitations of this study must be carefully considered (1) the major limitation of this meta-analysis was that tumor types included in this study were limited and the number of studies dealing with each type of cancers was ≤5, so that the results of the specific carcinomas might be less powerful; (2) the studies were almost retrospective and all were published in English, which was more susceptible to some biases; (3) the cutoff values of RDW were different; (4) studies lacking sufficient data were also excluded from the meta-analysis.

In conclusion, this is the first meta-analysis revealed that elevated RDW was an unfavorable predictor of prognosis in patients with cancers. However, due to the limitations uncovered in the present study, the results of our meta-analysis might be estimations. Future larger-scale prospective and standard investigations should be conducted to confirm these results.

## MATERIALS AND METHODS

### Literature collection

Two independently authors (LH, Hu and MM, Li) used the terms and combinations included: (“RDW” or “red blood cell distribution width”) and (“cancer” or “tumor” or ”carcinomas” or ”neoplasm”) and (prognosis or outcome or survival or mortality or recurrence or progression or metastasis) to identify studies in the databases of Web of Science, Embase, Pubmed and Cochrane Library, the detailed search strategy was included in supplementary files. The publication language was limited to English and the latest search was updated on July 15, 2016.

### Inclusion and exclusion criteria

The inclusion criteria are the following: (1) the role of RDW in cancer patients were investigated, (2) RDW was measured by blood-based methods without any formal treatment; (3) patients were divided into two groups according to cutoff values of RDW; (4) studies reported or containing sufficient data for the computation of hazard ratios (HR) and corresponding 95% confidence intervals (CI) for overall survival (OS) or (disease-free survival) DFS/(event-free survival) EFS/(progress-free survival) PFS/(cancer-specific survival) CSS.

The exclusion criteria are the following: 1) review, meeting abstract, letter, not full text in English; 2) duplicate publications; 3) nonhuman studies; 4) studies without usable data.Data extraction

Two independent authors (LH, Hu and MM, Li) extracted the following information from the eligible studies: first author, year of publication, study country, cancer type, sample size, cutoff value of RDW, and survival data. Disagreements were resolved by joint discussion.

### Quality assessment

The Newcastle-Ottawa Quality Assessment Scale (NOS) [37] was used to assess the quality of each study by two independent investigators (LH, Hu and MM, Li). The NOS contains three parts: selection (four points), comparability (two points), and outcome assessment (three points).

### Statistical analysis

The hazard ratio (HR) with 95% confidence intervals(95% CI) were directly obtained from the articles or estimate according to the method introduced by Tierney et al [38]. If there were more than one cutoff value of RDW, the HR and 95% CI belonging to the cutoff value which divided patients into the same size ratio were included. Get Data Graph Digitizer (http://getdata-graph-digitizer.com/ ) were used to obtain the data from the survival curve. Cochran Q test and I^2^ statistic were used to identify the heterogeneity among the included studies. If heterogeneity was significant (Cochran Q test: p value< 0.10 or I^2^> 50%), the random-effects model was used to estimate the pooled HR, and if not, the fixed-effects model was used. Publication bias was evaluated by using Begg's test and Egger's test. Trim-and-fill method was employed to further assess the possible effect of publication bias [39]. All statistical analyses were performed by using Stata 12 (Stata Corp., College Station, Texas) and P< 0.05 was considered statistically significant.

## SUPPLEMENTARY MATERIALS FILES



## Abbreviations

RDW: red blood cell distribution width
HR: hazard ratio
CI: confidence interval
OS: overall survival
DFS: disease-free survival
CSS: cancer-specific survival
EFS: event-free survival
PFS: progression-free survival
UTUC: upper tract urothelial carcinoma
CML: chronic myeloid leukemia
DLBCL: diffuse large B-cell lymphoma
ESCC: esophageal squamous cell carcinoma
