# Supplementary material for: Prognostic value of RDW in cancers: a systematic review and meta-analysis
Source: Oncotarget. 2016 Dec 2;8(9):16027–35. doi: 10.18632/oncotarget.13784 (PMC5362543; doi:10.18632/oncotarget.13784)
Supplement: Supplementary file 1 [file oncotarget-08-16027-s001.pdf]

Prognostic value of RDW in cancers: a systematic review and meta-analysis

Supplementary Material

Our search strategy

Pubmed: (((cancer or tumor or neoplasm or carcinoma[MeSH Terms])) AND (rdw or red blood cell distribution width[MeSH Terms])) AND (prognosis or outcome or survival or mortality or recurrence or progression or metastasis[MeSH Terms]) were used to search relevant studies.

Web of science: TOPIC: (cancer or tumor or neoplasm or carcinoma) AND TOPIC: (rdw or red blood cell distribution width) AND TOPIC: (prognosis or outcome or survival or mortality or recurrence or progression or metastasis) were used to search relevant studies.

WILEY: rdw or "red blood cell distribution width" in FullText AND cancer or tumor or neoplasm or carcinoma in FullText AND prognosis or outcome or survival or mortality or recurrence or progression or metastasis in Abstract were used to search relevant studies.

Embase: TITLE-ABSTR-KEY((rdw or "red blood cell distribution width")) and ALL((cancer or tumor or neoplasm or carcinoma) and (prognosis or outcome or survival or mortality or recurrence or progression or metastasis)) were used to search relevant studies in the journal section, and the article and short communication were chosen as type of journal.
